# Supplementary material for: Causal Relationship between Aging and Anorexia Nervosa: A White-Matter-Microstructure-Mediated Mendelian Randomization Analysis
Source: Biomedicines. 2024 Aug 16;12(8):1874. doi: 10.3390/biomedicines12081874 (PMC11351342; doi:10.3390/biomedicines12081874)
Supplement: Supplementary file 1 [file biomedicines-12-01874-s001.zip › Supplementary Figure/Figure S9/Leaveoneout plot of MR analysis for ubm-b-1820 to AN.pdf]

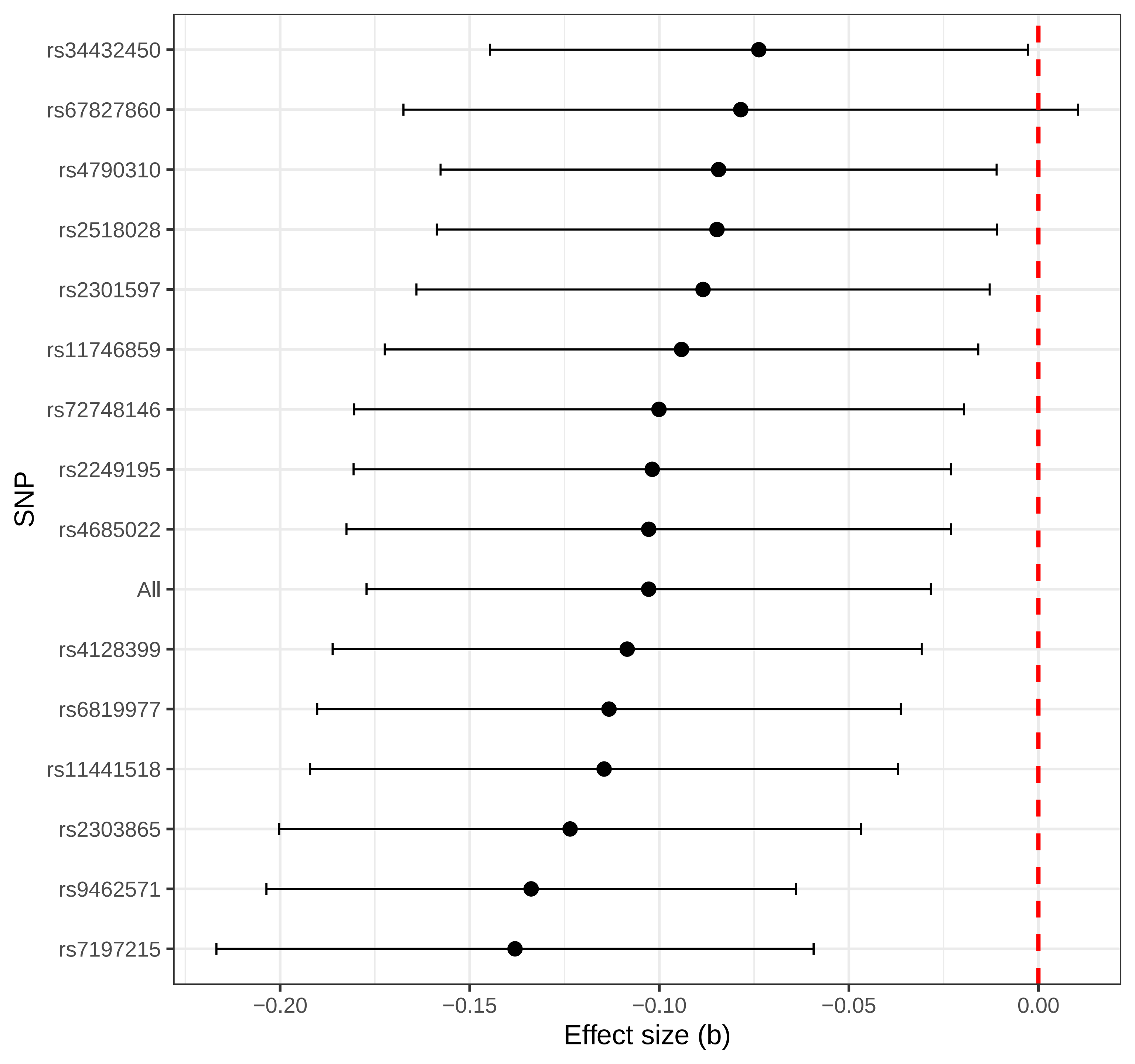

SNP
rs34432450
rs67827860
rs4790310
rs2518028
rs2301597
rs11746859
rs72748146
rs2249195
rs4685022
All
rs4128399
rs6819977
rs11441518
rs2303865
rs9462571
rs7197215
−0.20
−0.15
−0.10
−0.05
0.00
Effect size (b)
